# Multiple Valence Bands Convergence and Localized Lattice Engineering Lead to Superhigh Thermoelectric Figure of Merit in MnTe

**DOI:** 10.1002/advs.202206342

**Published:** 2023-04-24

**Authors:** Shahzada Zulkifal, Zhichao Wang, Xuemei Zhang, Suniya Siddique, Yuan Yu, Chong Wang, Yaru Gong, Shuang Li, Di Li, Yongsheng Zhang, Peng Wang, Guodong Tang

**Affiliations:** ^1^ MIIT Key Laboratory of Advanced Metallic and Intermetallic Materials Technology School of Materials Science and Engineering Nanjing University of Science and Technology Nanjing 210094 P. R. China; ^2^ National Laboratory of Solid State Microstructures College of Engineering and Applied Sciences and Collaborative Innovation Center of Advanced Microstructures Nanjing University Nanjing 210093 P. R. China; ^3^ Key Laboratory of Materials Physics Institute of Solid State Physics Chinese Academy of Sciences Hefei 230031 P. R. China; ^4^ Institute of Physics IA RWTH Aachen University 52056 Aachen Germany; ^5^ Advanced Research Institute of Multidisciplinary Sciences Qufu Normal University Qufu Shandong Province 273165 P. R. China

**Keywords:** dislocations, lattice thermal conductivity, localized lattice imperfections, multiple valence bands convergence, nanorods

## Abstract

MnTe has been considered a promising candidate for lead‐free mid‐temperature range thermoelectric clean energy conversions. However, the widespread use of this technology is constrained by the relatively low‐cost performance of materials. Developing environmentally friendly thermoelectrics with high performance and earth‐abundant elements is thus an urgent task. MnTe is a candidate, yet a peak *ZT* of 1.4 achieved so far is less satisfactory. Here, a remarkably high *ZT* of 1.6 at 873 K in MnTe system is realized by facilitating multiple valence band convergence and localized lattice engineering. It is demonstrated that Sb—Ge incorporation promotes the convergence of multiple electronic valence bands in MnTe. Simultaneously, the carrier concentration can be optimized by Sb—Ge—S alloying, which significantly enhances the power factor. Simultaneously, MnS nanorods combined with dislocations and lattice distortions lead to strong phonon scattering, resulting in a markedly low lattice thermal conductivity(*κ*_lat_) of 0.54 W m K^−1^, quite close to the amorphous limit. As a consequence, extraordinary thermoelectric performance is achieved by decoupling electron and phonon transport. The vast increase in *ZT* promotes MnTe as an emerging Pb‐free thermoelectric compound for a wide range of applications in waste heat recovery and power generation.

## Graphical abstract

An ultra‐high figure of merit (*ZT*) is realized in MnTe via multiple valence bands convergence and localized lattice engineering by Sb—Ge—S alloying. The significant increase in *ZT* indicates MnTe as an emerging lead‐free thermoelectric material system for wide range of applications in waste heat recovery and power generation.

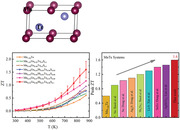

## 1 Introduction

Thermoelectric materials enabling a reversible conversion between thermal energy and electrical power have been considered a promising alternative to meet the challenges of the global energy dilemma.^[^ ^1^, ^2^, ^3^ ^]^ The conversion efficiency of thermoelectric material is quantized by the dimensionless figure of merit *ZT*, *ZT* = *S* ^2^ *σT*/*κ*_T_, where *S*, *σ*, *κ*_T_, and *T* are the Seebeck coefficient, electrical conductivity, total thermal conductivity, and absolute temperature, respectively. *κ*_T_ consists of lattice thermal conductivity (*κ*_lat_) and electronic thermal conductivity (*κ*_ele_).^[^ ^4^, ^5^, ^6^ ^]^ High‐performance thermoelectric materials require a high *ZT* value, which is often encountered by a number of interconnected challenges. Engineering carrier concentration, modifying electronic band structures,^[^ ^2^, ^7^, ^8^ ^]^ reducing thermal conductivity by designing multiscale microstructures,^[^ ^9^ ^]^ looking for materials with intrinsically low thermal conductivity,^[^ ^10^, ^11^ ^]^ and decoupling electron and phonon transport,^[^ ^12^ ^]^ are some strategies developed to improve the thermoelectric performance in different thermoelectric systems. Benefitting these strategies, PbTe‐based materials,^[^ ^13^, ^14^, ^15^ ^]^ skutterudites,^[^ ^16^ ^]^ half‐Heusler alloys,^[^ ^17^ ^]^ Mg_2_Si,^[^ ^18^ ^]^ SnSe,^[^ ^8^, ^19^ ^]^ and GeTe^[^ ^20^ ^]^ has been extensively explored as efficient mid‐temperature thermoelectric systems.

Chalcogenide MnTe has attracted considerable interest as an emerging promising mid‐temperature thermoelectric candidate due to its lead‐free nature, high content of Mn in the earth's crust, and superior mechanical properties.^[^ ^8^, ^9^ ^]^ MnTe crystallizes with a typical hexagonal NiAs crystal structure (space group of *P*63/*mmc*) without involving phase transition at elevated temperature.^[^ ^23^ ^]^ However, unlike some other *p*‐type metal telluride such as PbTe, SnTe, and GeTe with narrow band gaps, the thermoelectric performance of MnTe is inferior and unimpressive. Compared with GeTe and PbTe, the electronegativity difference between Mn (1.55) and Te (2.1) is larger. In general, the larger electronegativity difference between the elements gives rise to the increased polarity of the bond. The increase of bond polarity usually leads to the strong scattering of polar optical phonons to carriers, resulting in low carrier mobility. Besides, MnTe is a broadband gap semiconductor with an indirect band gap of 0.86 eV and a direct band gap of 1.27 eV,^[^ ^24^ ^]^ leading to the low intrinsic carrier concentration. The culprits of the low *ZT* value of MnTe are manifold. First, the intrinsic carrier concentration (10^18^ cm^−3^) of MnTe is far from optimization.^[^ ^22^ ^]^ The substantial, significant electronegativity difference between Mn (1.55) and Te (2.10) causes strong charge carrier scattering by optical phonons, which is detrimental to carrier mobility.^[^ ^25^ ^]^ Its low carrier mobility (6 cm^2^ V^−1^ s^−1^) and low carrier concentration limit its electrical transport properties in pristine MnTe.^[^ ^22^ ^]^

The optimization of electrical transport properties with less sacrifice of other thermoelectric parameters can significantly improve the thermoelectric performance of MnTe. In 2013, Kim et al.^[^ ^26^ ^]^ reported non‐stoichiometric Mn_0.51_Te_0.49_ with a *ZT* value of 0.41 at 773 K. Chemical doping/substitution can optimize the carrier concentration and lead to an enhancement of the electrical conductivity. Substituting monovalent metals (such as Ag,^[^ ^27^ ^]^ Cu,^[^ ^28^ ^]^ Sb,^[^ ^29^ ^]^ Li,^[^ ^30^ ^]^ Na^[^ ^21^ ^]^) for Mn and incorporating inclusions with high electrical conductivities (such as Ag_2_S,^[^ ^31^ ^]^ SnTe,^[^ ^32^ ^]^ Sb_2_Te_3_^[^ ^33^ ^]^) have been explored promising ways for improving its electrical conductivity. Xie et al.^[^ ^25^ ^]^ successfully substituted Sulfur (S) for Te anion sites to modulate the electrical conductivity of MnTe and reported a *ZT* of 0.65 at 773 K in MnTe_0.9_S_0.1_. In many cases, the Seebeck coefficient is seriously deteriorated by enhanced carrier concentration, which significantly limits the ability to optimize electrical transport properties and *ZT* value further.^[^ ^34^ ^]^ Therefore, decoupling and synergistically optimizing the *S* and σ are greatly desired for achieving high‐performance MnTe‐based thermoelectric materials.^[^ ^32^ ^]^ Band convergence is an appealing route to accomplish a significant *S* while maintaining high σ.^[^ ^2^ ^]^ It is challenging to attain multiple valence band convergence in MnTe. Ge and Sb dopants possess large differences in atom radii with host Mn element, which can introduce mass fluctuation and possibly lattice imperfections into MnTe matrix, leading to reduced *κ*_lat_. The electronegativity difference between Ge (2.01), Sb (2.05), and Te (2.10) is smaller than that of Mn (1.55), benefiting the electrical transport properties. Sulfur (S) for Te anion sites to modulate the σ of MnTe has been reported.^[^ ^25^ ^]^ This motivates us to optimize thermoelectric performance of MnTe by Ge—Sb—S alloying.

In this work, we demonstrate that *κ*_lat_ and record‐high thermoelectric performance *ZT* of 1.6 were achieved in the MnTe system through localized lattice engineering and facilitating multiple valence band convergence. Convergence of multiple electronic valence bands promoted by Sb—Ge incorporation, coupled with enhanced electrical conductivity due to optimized carrier concentration, produces a sharp increase in power factor (PF). Moreover, ultralow lattice thermal conductivity is obtained thanks to localized lattice imperfections, including MnS nanorods, dislocations and lattice distortions induced by Sb—Ge—S and alloying with MnTe. The high thermoelectric performance shows the high potential of MnTe for thermoelectric power generation at medium temperatures.

## 2 Experimental Section

The high‐quality and homogenous polycrystalline ingots with the stoichiometry Mn_1.06‐x_Ge_x_Te_0.9_S_0.1_ (x = 0.05 and x = 0.08) and Mn_1.06‐x‐y_Ge_x_Sb_y_Te_1‐z_S_z_ are prepared by a combined melt‐quenching method and spark plasma sintering (SPS). The high‐purity Mn (99.9%), Sb (99.999%), Te (99.99%), Ge (99.99%), and S (99.9%) powders were used as the starting materials. The incorporation of extra Mn tends to induce more V_Te_ or Mn_Te_.^[^ ^35^ ^]^ Likewise, extra Mn effectively occupies interstitial sites, suppressing thermal conductivity.^[^ ^36^ ^]^ The powders were mixed in an agate mortar and loaded in a quartz tube. The powder was melted at 1273 K for 50 h and cooled down to room temperature through water quenching. Then, obtained ingots of pristine Mn_1.06_Te and Ge—Sb—S doped Mn_1.06_Te were crushed and ground into powders again in a mortar‐pestle and then consolidated by Spark Plasma Sintering (SPS) at 973 K for 6 min under an axial pressure of 50 MPa.

The obtained bulk samples were then used to measure the phase structures using an X‐ray diffraction (XRD) instrument (Bruker D8 Advance) equipped with Cu K*α* radiation. The morphology and microstructure of the samples were confirmed using a High‐Resolution Scanning Electron Microscope Gemini SEM 500. The inside Energy Dispersive Spectrometer (EDS) was used to obtain the elemental mapping of the sample. An FEI Titan3 G2 60–300 STEM equipped with a double aberration corrector was used to perform high‐angle annular dark field scanning transmission electron microscopy (HAADF‐STEM) imaging and energy dispersive X‐ray spectroscopy (EDS) mapping at 300 kV. Samples used for observation were prepared by focused ion beam (FIB) milling using the in situ lift‐out technique on a FEI Nova Nanolab DualBeam instrument. The electrical resistivity (*ρ*) and Seebeck coefficient (*S*) were measured simultaneously using the Ulvac‐Riko ZEM‐3 instrument system under a helium atmosphere from 300 to 873 K. The laser flash diffusivity method (Netzsch, LFA 457, Germany) was applied to determine the thermal diffusivity (*D*) in an argon protection environment. Both the electrical and thermal transport properties were measured along the pressing direction for dense pellets. The specific heat capacity (*C*_p_) was derived from the previous study.^[^ ^22^ ^]^ The sample density (*ρ*) was determined by a density meter (ME204E) using the Archimedes method. The total thermal conductivity (*κ*) is derived using the formula *κ* = *DC*_p_ *ρ*. Thermal and electrical transport properties were measured along the pressing direction. The Hall‐carrier concentration (*n*_H_) and Hall‐mobility (*µ*) were determined using Hall measurement instrument (HMS‐3000) by the Van der Pauw method. UV–vis–NIR absorption spectrum measurements were carried out to determine the band gap using Shimadzu UV‐3600i Plus. The uncertainty of the Seebeck coefficient (*S*) and electrical conductivity (*σ*) measurement is within ≈5%. The uncertainty of the thermal conductivity is to be within ≈12%, considering the uncertainties of ≈5% for diffusivity (*D*), ≈5% for specific heat (*C*_p_) and ≈2% for sample density *(ρ*). The combined uncertainty for all measurements involved in the calculations of *ZT* is ≈ 20%.

**Figure 1:**
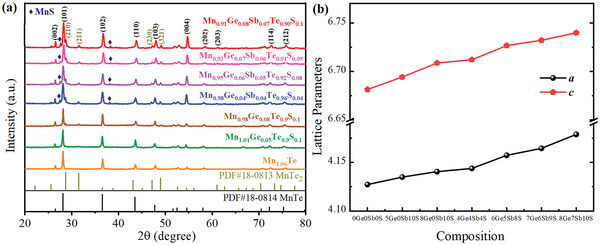
a) XRD patterns, and b) lattice parameters of Mn_1.06‐x_Ge_x_Te_0.9_S_0.1_ and Mn_1.06‐x‐y_Ge_x_Sb_y_Te_1‐z_S_z_ samples.

The density functional theory calculations utilizing the projector augmented wave (PAW) method are performed using the Vienna ab initio simulation package (VASP).^[^ ^37^ ^]^ The generalized gradient approximation (GGA) of Perdew, Burke, Ernzerhof (PBE) is used for the electronic exchange‐correlation (EXC) function.^[^ ^38^ ^]^ In order to calculate the MnTe system, spin‐polarized PBE+ U calculations (U = 4.8 eV)^[^ ^39^ ^]^ are used to determine the electronic properties of the compound. The cutoff energy for plane‐wave expansion of the wave functions is 450 eV, and the total energy is converged to within 10^−5^ eV. Especially the spin‐polarization is included in the MnTe compound. To elucidate the effects of S, Ge and Sb alloying on the electrical properties of MnTe, we construct a large (4 × 4 × 2) MnTe supercell (*a* = 16.58 Å, b = 16.58 Å, c = 13.42 Å, containing 64 cations and 64 anions) based on the experimentally suggested doping concentrations. On the one hand, S/Ge/Sb alloying is too complex on the simulation side; on the other hand, to understand the effect of cation (Ge, Sb) and anion (S) alloying on the band structures, we separate the three dopes into two parts, the S anion single alloying and Ge/Sb cation alloying. Taking the experimentally suggested alloying concentrations of S (10%), Ge (8%) and Sb (7%) with the highest *ZT* values, we build the MnTe_0.891_S_0.109_ and Mn_0.844_Ge_0.078_Sb_0.078_Te supercells to simulate the effect of electronic properties of ≈10% S single alloying and 7–8% Ge/Sb alloying in MnTe, respectively. All possible configurations of S, and Ge/Sb in the MnTe matrix have been considered (the detailed configuration construction in Supporting Information), and we choose the most stable configurations in Figure S1 (Supporting Information). For the Brillouin zone (BZ) integrations, the Monkhorst–Pack.^[^ ^40^ ^]^ k‐point meshes of (15 × 15 × 10) and (3 × 3 × 3) are used for the pristine and supercell MnTe systems, respectively. The geometry structures are fully optimized until all the forces and components of the stress tensor are < 0.01 eV Å^−2^ and 0.2 kbar, respectively. Since the supercell is established according to the structure of a primitive cell, band folding is found in the band structure of a supercell. Then, we apply a band unfolding methodology (the Band UP code) along the high symmetry directions of the primitive cell and then recover the effective primitive picture.^[^ ^41^ ^]^ Beside, the lattice parameter increasement is negligible, only ≈0.8% for the largest one. Such small lattice distortion has little effects on the electronic structures (**Figure** **1**). Thus, in the supercell defect DFT simulations, we fix the lattice constants of supercell and fully relax the atomic positions as in Refs. [42, 43, 44] Only the effects of atomic geometry relaxations not the lattice distortion are considered in the electronic structures.

## 3 Results and Discussion

Figure 1a shows the X‐ray diffraction (XRD) patterns of Mn_1.06‐x_Ge_x_Te_0.9_S_0.1_ and Mn_1.06‐x‐y_Ge_x_Sb_y_Te_1‐z_S_z_ samples. All the major diffraction peaks can be indexed to the hexagonal MnTe structure (PDF# 18–0814) with space group *P*63/*mmc*. Additional peaks of the MnTe_2_ secondary phase can be found in all doped Mn_1.06‐x_Ge_x_Te_0.9_S_0.1_ and Mn_1.06‐x‐y_Ge_x_Sb_y_Te_1‐z_S_z_ samples. SEM and EDS mapping (Figure S2, Supporting Information) confirm the presence of MnTe_2_ phase. An increase of Sb content beyond 1.5% is reported to result in MnTe_2_ secondary phase in Mn_1‐x_Sb_x_Te samples, which is due to the solid solubility limit of Sb into MnTe.^[^ ^29^ ^]^ MnTe_2_ impurity phase is also probably due to the oxidization during the storage and high temperature synthesis procedures.^[^ ^27^ ^]^ Moreover, trace amount of MnS secondary phase are observed in all samples of Mn_1.06‐x‐y_Ge_x_Sb_y_Te_1‐z_S_z_, which indicates the sulfur doping content has already exceeded the solubility limit. The shift of the diffraction peaks toward lower angles (Figure S3, Supporting Information) with the increase of doping content implies the presence of lattice expansion. The calculated lattice parameters *a* and *c* increase with an increase of alloying contents, as shown in Figure 1b. There are two possible sites for heterogeneous atoms doping into the lattice of MnTe: Sb and Ge substitute the Mn site, and S substitutes the Te site. The ionic radii of Sb (206 pm) and Ge (125 pm) are larger than that of Mn (67 pm), whereas the ionic radius of S (184 pm) is smaller than that of Te (221 pm). Thus, the lattice expansion can be attributed to the more dominant contribution of Sb and Ge over that of S alloying. SEM investigations were conducted on the polished surface of Mn_0.91_Ge_0.08_Sb_0.07_Te_0.90_S_0.1_ sample. As shown in Figure S4a (Supporting Information), the polished surface of the sample contains various areas with distinctive contrast. Elemental mapping of individual elements (Figure S4b–f, Supporting Information) indicates Mn and S accumulation, identifying the existence of MnS precipitates. Although S is precipitated as the MnS phase, most of S still forms the solid solution in the MnTe matrix, as shown in Figure S4f (Supporting Information). We calculate the defect formation energies of MnTe_0.891_S_0.109_, Mn_0.922_Ge_0.078_Te, Mn_0.922_Sb_0.078_Te and Mn_0.844_Ge_0.078_Sb_0.078_Te (Table S5, Supporting Information). We find that the defect formation energy of S doping is negative (−0.03 eV), which means that the S would forms new phase with Mn. It is consistent with the experimentally observed MnS precipitate. However, for Ge and Sb single or co‐doping, the not too high positive defect formation energies (0.39 eV per defect for the Ge/Sb co‐doping) suggest that they have the solid solution probability.

**Figure 2:**
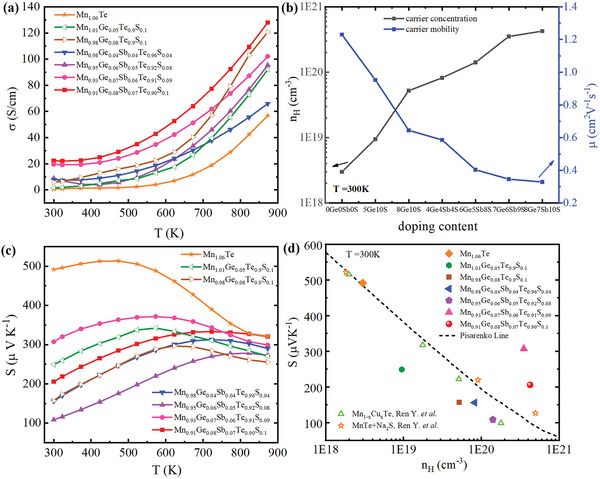
Thermoelectric properties of Mn_1.06‐x_Ge_x_Te_0.9_S_0.1_ and Mn_1.06‐x‐y_Ge_x_Sb_y_Te_1‐z_S_z_ samples as a function of temperature: a) electrical conductivities (*σ*), b) compositional dependence of carrier concentration (*n*_H_) and carrier mobility (*µ*) at room temperature, c) Seebeck coefficients (*S*), d) the carrier concentration dependent Seebeck coefficients of samples at room temperature.

The temperature dependence of*σ* for Mn_1.06‐x_Ge_x_Te_0.9_S_0.1_ and Mn_1.06‐x‐y_Ge_x_Sb_y_Te_1‐z_S_z_ samples is shown in **Figure** **2**a. *σ* first behaves like a plateau < 523 K originating from the scattering of majority carriers^[^ ^33^ ^]^ and then increases sharply > 523 K due to the intensive supplement of carrier concentration induced by the well‐known intrinsic excitation.^[^ ^25^ ^]^ Notably, Mn_1.06_Te has a low electrical conductivity of 0.97 S cm^−1^ at room temperature owing to its low carrier concentration and mobility. Compared with pristine Mn_1.06_Te, Mn_1.06‐x_Ge_x_Te_0.9_S_0.1_ and Mn_1.06‐x‐y_Ge_x_Sb_y_Te_1‐z_S_z_ samples exhibit significantly enhanced *σ* in the whole temperature range. For Mn_1.06‐x_Ge_x_Te_0.9_S_0.1_ samples *σ* is enhanced with the increasing Ge doping content. Besides, by introducing Sb into Ge—S alloyed samples, *σ* exhibit a distinct increase in the investigated temperature range. Specifically, *σ* dramatically increases from 0.58 S cm^−1^ for pristine Mn_1.06_Te to ≈ 23 S cm^−1^ for Mn_0.91_Ge_0.08_Sb_0.07_Te_0.9_S_0.1_ at room temperature. Typically, the highest *σ* of 124.24 S cm^−1^ is obtained for Mn_0.91_Ge_0.08_Sb_0.07_Te_0.9_S_0.1_ at 873 K. The increase in *σ* has a close relationship with the increase in carrier concentration (*n*_H_). To determine the hole carrier concentration, Hall measurements are carried out, and the results are presented in Figure 2b. Clearly, *n*_H_ elevates rapidly with increasing Sb—Ge—S alloying content. For Ge and S codoped Mn_1.06‐x_Ge_x_Te_0.9_S_0.1_, *n*_H_ increases with Ge doping level, which reveals that Ge can enhance *n*_H_. The electronegativity difference between Ge (2.01), and Te (2.10) is smaller than that of Mn (1.55), benefiting the electrical transport properties. The small electronegativity differences lead to the formation of covalent bonding between Ge and Te. The resulting localization of electrons will be weakened such that the electrons can jump into conduction bands more easily, which can contribute to enhance the carrier concentration.^[^ ^45^ ^]^ Sb doping can further improve *n*_H_, as shown in Figure 2b. Sulphur doping is reported to elevate electrical conductivity to some extent.^[^ ^25^ ^]^ Ge and Sb substitution have been noted the dominant contributor to the improved *n*_H_ as well as *σ*. At the same time, the Hall mobility *µ* decreases due to the induced impurity scattering by dopants. Thus, the increase in *σ* could be attributed to the increased carrier concentration, despite the slight decrease in Hall mobility. Compared to undoped Mn_1.06_Te, *σ* of Mn_1.06‐x‐y_Ge_x_Sb_y_Te_1‐z_S_z_ compounds is significantly optimized because of boosted carrier concentration. Figure 2c describes the Seebeck coefficient (*S*) temperature dependence for Mn_1.06‐x_Ge_x_Te_0.9_S_0.1_ and Mn_1.06‐x‐y_Ge_x_Sb_y_Te_1‐z_S_z_ compounds. The positive Seebeck coefficient indicating the *p*‐type conduction is in good agreement with Hall measurements. *S* increases with temperature, reaching a maximum value at a certain temperature, and then slightly decreases at elevated temperature, indicating degenerate semiconductor behaviour. The Seebeck peak (*S*_max_) suggests the onset of intrinsic excitation, that is, bipolar carrier diffusion.^[^ ^46^ ^]^ However, the Seebeck peaks of doped MnTe shift to a higher temperature compared with that of undoped Mn_1.06_Te, which are due to the widening band gap (illustrated in **Table** **1**). Mn_1.06‐x‐y_Ge_x_Sb_y_Te_1‐z_S_z_ compounds show reduced *S* than undoped Mn_1.06_Te. The well‐established Pisarenko relation between the *S* and*n*_H_ reveals some valuable information about the electronic band structure changes. It thus has a deeper insight into the electrical transport properties, as shown in Figure 2d. The black dashed line is the theoretical Pisarenko line corresponding to a single parabolic band (SPB) with the assumption of acoustic phonon scattering.^[^ ^47^ ^]^ The carrier‐concentration‐dependent Seebeck coefficient of Mn_1.06‐x_Ge_x_Te_0.9_S_0.1_ and Mn_1.06‐x‐y_Ge_x_Sb_y_Te_1‐z_S_z_ samples is compared with previously reported data of Na_2_S^[^ ^21^ ^]^ and Cu^[^ ^28^ ^]^ doped MnTe in Figure 2d at room temperature. The measured data of pristine Mn_1.06_Te from our study and those of doped MnTe samples in reported literature lie on the Pisarenko line, indicating the validity of the SPB model for MnTe and revealing that a traditional doping behaviour makes little contribution to the band structure of MnTe. The Seebeck coefficient of Mn_0.91_Ge_0.08_Sb_0.07_Te_0.9_S_0.1_ and Mn_0.93_Ge_0.07_Sb_0.06_Te_0.91_S_0.09_ lies above the Pisarenko line, indicating the modified band structure caused by Sb—Ge—S doping.

**Table 1:** Band gap and energy differences between the four valence maxima (the Γ, M, H, and A points in the BZ)

eV	MnTe [PBE]	MnTe_0.891_S_0.109_	Mn_0.844_Ge_0.078_Sb_0.078_Te
Band gap	0.71	0.83	0.81
Δ*ε*_Γ‐M_	0.04	0.08	0.02
Δ*ε*_Γ‐H_	0.26	0.25	0.24
Δ*ε*_Γ‐A_	0.34	0.35	0.23

**Figure 3:**
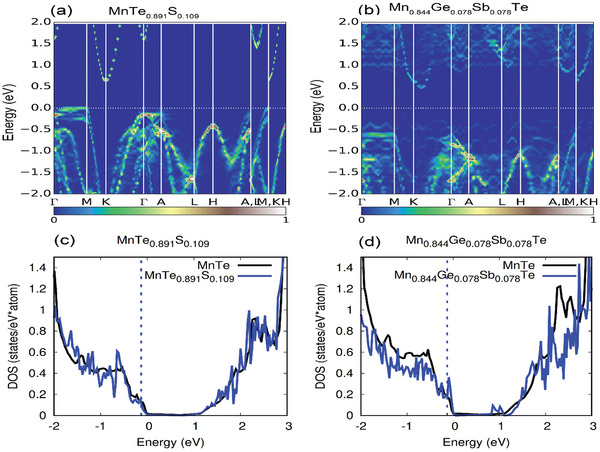
Electronic band structures of a) MnTe_0.891_S_0.109_ and b) Mn_0.844_Ge_0.078_Sb_0.078_Te. The scale bar is the magnitude of the spectral weight, which characterizes the probability of the primitive cell eigenstates contributing to a particular supercell eigenstates of the same energy, the color bar represents the magnitude of the spectral weight, which characterizes the probability of the primitive cell eigenstates contributing to a particular supercell eigenstate of the same energy. Electronic density of states of c) MnTe_0.891_S_0.109_ and d) Mn_0.844_Ge_0.078_Sb_0.078_Te, the blue dashed lines in the density of states represent the Fermi level.

To understand the effect of Sb, Ge and S doping on the electrical properties of MnTe, we calculate their electronic band structures by density functional theory calculation. Due to the well‐known underestimated band gap in the PBE calculations, we follow the strategy used in Ref. [32] and correct the PBE‐calculated MnTe band structures using the HSE calculations. Thus, from the fixed band structures of pristine MnTe compound (Figure S5, Supporting Information), the band gap is 0.71 eV and the energy differences of valence band maxima of ΔE(Γ‐M)_,_ ΔE(Γ‐H) and ΔE(Γ‐A) are 0.04, 0.26 and 0.34 eV in Table 1, respectively, which are very close to the HSE results in Ref. [32] To carry out band engineering of the *p*‐type MnTe, the contributions of atomic levels on valence band maximum (VBM) should be revealed. From the projected density of states (PDOS) and crystal orbital Hamilton population (COHP) of MnTe (Figure S6, Supporting Information), we notice that the VBM is dominated by the antibonding region between the Mn‐d and Te‐p orbitals. When alloying10% anion S in MnTe, its unfoldering band structures and density of states (DOS) are given in **Figure** **3**. We find that the band features (Figure 3a) and the DOS (Figure 3c) near the VBM of 10% S doped MnTe are similar as those in the pristine MnTe compound (Figure S5, Supporting Information): the energy differences between the valence band maxima (at the Γ, M, H and A points in Figure 3a) are nearly no changing (Table 1). Only the band gap with S alloying is increased to 0.83 eV. This suggests that introducing S in the MnTe matrix barely contributes to the electrical transport properties of the MnTe system. For the Ge or Sb doped MnTe (Mn_0.922_Ge_0.078_Te and Mn_0.922_Sb_0.078_Te, (Figure S7, Supporting Information), although the band gaps of Ge or Sb doping are increasing to suppress the possible bipolar effects (Table S3, Supporting Information), the band convergence is not comparable to those in the Ge—Sb co‐doping case. The Ge doping does not contribute the band convergence at all; The energy difference ΔE(Γ‐M)_,_ ΔE(Γ‐H) and ΔE(Γ‐A) are nearly the same as those in the pristine MnTe. For the Sb doping, the band convergence is only slightly increased. For the Ge/Sb cation codoping, on the other hand, we notice that the energy differences between the valence band maxima are changed obviously (Figure 3b). At the 7.8%Ge—Sb in MnTe (Mn_0.844_Ge_0.078_Sb_0.078_Te), the energy difference (Table 1) of ΔE(Γ‐M), ΔE(Γ‐H) and ΔE(Γ‐A) decreases to 0.02, 0.24 and 0.23 eV, respectively, which suggests the band convergence among the bands at Γ, H and A high symmetry points (Figure 3b). Generally speaking, the Ge and Sb doping do show the band convergency behavior (Figure S7 and Table S3, Supporting Information), but are only favorable for the bands of ΔE(Γ‐H) (deceased to 0.24 eV) and ΔE(Γ‐A) (deceased to 0.28 eV), respectively. However, the Ge/Sb co‐doping can take advantage such convergency behavior, and simultaneously converge ΔE(Γ‐H) and ΔE(Γ‐A). This can be understood by the significant change of the interactions between the cation and anion. In the pristine MnTe compound, we already know that the VBM is contributed by the antibonding states between the Mn‐d and Te‐p orbitals. Since the d orbital is localized, the interactions between Mn‐d and Te‐p are strong, and the VBM is pushed to a high‐energy position. When substituting Mn by Ge and Sb, their p orbitals are close to that of Te‐p (Table S3, Supporting Information), and the orbital interactions at the VBM are now changed to the antibonding states between Ge/Sb‐p and Te‐p. Since the Ge/Sb‐p orbital is more delocalized than that of Mn‐d, the interactions between Ge/Sb‐p and Te‐p is weaker than those of Mn‐d and Te‐p. This will lower the energy position of the antibonding state VBM and lead to band convergence. The strong band convergence between the valence maxima would suggest the high DOS (a peak just below the VBM in Figure 3d) and the high *S*. From the density of states (DOS) of Ge/Sb co‐doping (Figure 3d), the Fermi level position is close to the valence band maximum (VBM), and the contribution of the band convergence on the transport properties is important. The band gap also increases to 0.81 eV, which will also suppress the possible bipolar effect. This is consistent with the experimentally observed significantly enhanced Seebeck coefficient in MnTe‐8%Ge‐7%Sb. Our DFT calculations show that introducing Ge—Sb codoping would facilitate multiple valence band convergence and produce high DOS values in the electronic structure of MnTe, leading to the enhancement of the *S*.

**Figure 4:**
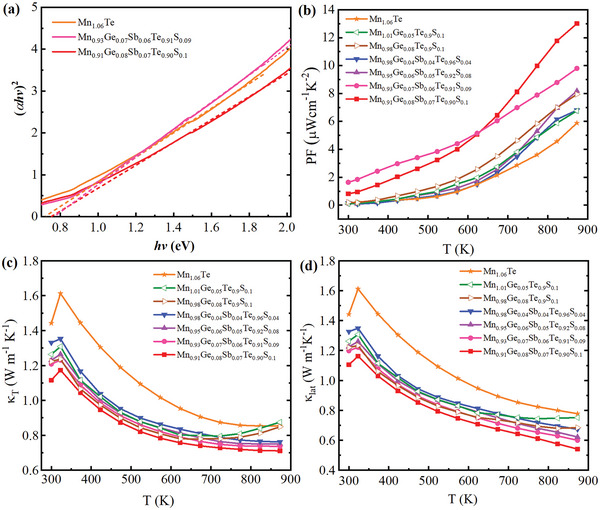
a) (*αhν*) ^2^‐*hν* plot showing the band gap of Mn_1.06_Te, Mn_0.93_Ge_0.07_Sb_0.06_Te_0.91_S_0.09_ and Mn_0.91_Ge_0.08_Sb_0.07_Te_0.9_S_0.1_. The temperature dependent b) Power factor (*PF*), c) total thermal conductivity (*κ*_T_), d) lattice thermal conductivity (*κ*_lat_) of Mn_1.06‐x_Ge_x_Te_0.9_S_0.1_ and Mn_1.06‐x‐y_Ge_x_Sb_y_Te_1‐z_S_z_ samples.

The band gap of Mn_1.06‐x‐y_Ge_x_Sb_y_Te_1‐z_S_z_ samples is measured using UV–vis–NIR absorption spectra measurements to experimentally confirm the band gap modification caused by incorporating Sb—Ge—S to MnTe. The band gap (*E*_g_) value is estimated from the (*αhv*)^2^–*hv* plot, where *α* is the absorption coefficient, *h* is the Planck's constant, and *v* is the photon frequency as illustrated in **Figure** **4**a. The band gap value is found at the point of intersection with the *x*‐axis when the linear portion of (*αhv*)^2^–*hv* plot is extended. The band gap for Mn_1.06_Te Mn_0.93_Ge_0.07_Sb_0.06_Te_0.91_S_0.09_ and Mn_0.91_Ge_0.08_Sb_0.07_Te_0.9_S_0.1_ is 0.73, 0.76, and 0.78 eV, respectively. Mn_1.06‐x‐y_Ge_x_Sb_y_Te_1‐z_S_z_ samples have larger band gap than Mn_1.06_Te, the experimental results are compatible with the theoretical explanation. The temperature‐dependent power factor (*S* ^2^ *σ*) for Mn_1.06‐x_Ge_x_Te_0.9_S_0.1_ and Mn_1.06‐x‐y_Ge_x_Sb_y_Te_1‐z_S_z_ samples is shown in Figure 4b. The *PF* is significantly enhanced through Ge—Sb—S incorporation. We obtain the maximum power factor of 7.94 µW cm^−1^ K^−2^ in Mn_0.98_Ge_0.08_Te_0.9_S_0.1_ sample at 873 K. Whereas, the maximum *PF* of 12.89 µW cm^−1^ K^−2^ at 873 K is realized in Mn_0.91_Ge_0.08_Sb_0.07_Te_0.9_S_0.1_ at 873 K, which is improved by ≈ 120% compared with that of pristine Mn_1.06_Te (5.87 µW cm^−1^ K^−2^). Ge and Sb alloying induce high multiple valence band convergence in the electronic structure of MnTe, producing an enhanced Seebeck coefficient. The enhanced Seebeck coefficient coupled with dramatically improved carrier concentration results in the sharp increase of *PF* in Mn_1.06‐x‐y_Ge_x_Sb_y_Te_1‐z_S_z_ series.

The*κ*_T_ for Mn_1.06‐x_Ge_x_Te_0.9_S_0.1_ and Mn_1.06‐x‐y_Ge_x_Sb_y_Te_1‐z_S_z_ series as a function of temperature are presented in Figure 4c. *κ*_T_ is significantly suppressed compared to that of undoped Mn_1.06_Te. MnTe is proposed to exhibit magnetic phase transition at 310 K that contributes to different electrical and thermal transport properties at low temperatures. The observed peak variation in thermal conductivity at low temperature is due to the phase transition at Neel's temperature.^[^ ^48^ ^]^ Mn_0.91_Ge_0.08_Sb_0.07_Te_0.9_S_0.1_ specimen exhibits the lowest *κ*_T_ among all investigated samples. Above 700 K, *κ*_T_ of Mn_1.06‐x_Ge_x_Te_0.9_S_0.1_ samples is observed to be slightly increased with temperature, which can be attributed to dramatical increase in *σ*, leading to the larger contribution of *κ*_ele_ at high temperatures. The *κ*_ele_ has been derived based on the Wiedemann–Franz law *κ*_ele_ *= LσT* that is demonstrated in Figure S8 (Supporting Information). The Lorenz number (*L*) was derived by the fitting of respective Seebeck coefficient values with an assumption of a single parabolic band model,^[^ ^49^ ^]^ and only acoustic phonon scattering considered (Figure S9, Supporting Information). *κ*_ele_ increases in doped samples due to enhanced carrier concentration. The *κ*_lat_ is obtained after subtracting *κ*_ele_ from *κ*_Τ_. *κ*_lat_ of Mn_1.06‐x_Ge_x_Te_0.9_S_0.1_ and Mn_1.06‐x‐y_Ge_x_Sb_y_Te_1‐z_S_z_ samples reduces as compared to undoped Mn_1.06_Te, as illustrated in Figure 4d. Sb alloying in addition to Ge and S can effectively suppress the *κ*_lat_, particularly at high temperatures. The decrease in *κ*_lat_ is mainly attributed to the extra phonon scattering, originating from the alloy scattering, mass fluctuation and strain field fluctuation by point defects introduced through Ge and Sb substitution on Mn sites. Mn_0.91_Ge_0.08_Sb_0.07_Te_0.9_S_0.1_ exhibits the lowest *κ*_lat_ among all doped samples. The lowest *κ*_lat_ of 0.54 W m^−1^ K^−1^ is achieved at 873 K for this composition.

**Figure 5:**
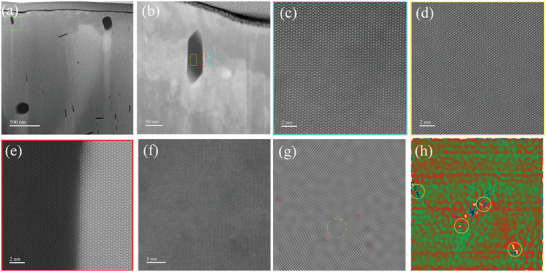
Microstructural characterization of Mn_0.91_Ge_0.08_Sb_0.07_Te_0.90_S_0.1_: a) typical HAADF‐STEM image showing the distribution of plenty of nanoprecipitates and nanorods, b) an enlarged view of the green rectangle in Figure 5a, c,d) atomic resolution HAADF‐STEM image of MnTe matrix and nanoprecipitates, respectively, e) lattice image depicts the interface between the nanoprecipitate and matrix phase, f) the corresponding image showing high‐density of dislocations and lattice distortions, g) filtered image based on (e) showing dislocations (marked with T) and lattice distortions (encircled), h) phase strain distribution map of (g).

**Figure 6:**
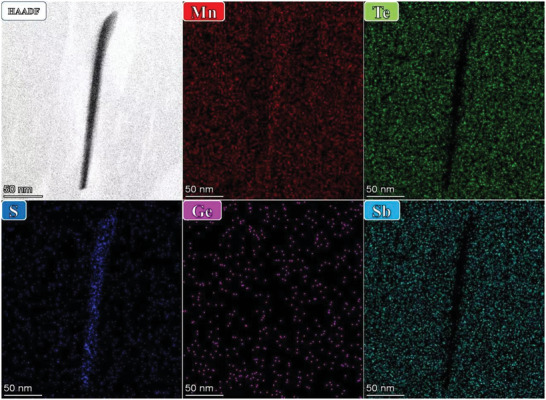
STEM‐EDS elemental mapping of nanorods revealing the composition of these precipitated phase is mainly Mn and S elements.

Microstructural characterizations were performed on Mn_0.91_Ge_0.08_Sb_0.07_Te_0.9_S_0.1_ sample using an FEI Titan G2 60–300 STEM equipped with a double aberration corrector for elucidating the underlying mechanism of reduced *κ*_lat_. Low magnification HAADF‐STEM image reveals that two main types of nanoprecipitates are observed. As shown in **Figure** **5**a, high‐density nanorods with an average size of ≈ 10 nm were found in the MnTe matrix. In addition, nanoprecipitates appearing as dark quasi‐circular (oval) and elongated shapes with 150–200 nm in size can be observed. Mn and S accumulation in STEM‐EDS elemental mapping indicates that both nanorods (**Figure** **6**) and nanoprecipitates (Figure S11, Supporting Information) are MnS phase. The contrast of the HAADF‐STEM image is monotonically proportional to the atomic number.^[^ ^50^, ^51^, ^52^ ^]^ Nanorods and nanoprecipitates appear in dark contrast (Figure 5a), confirming the dark domain as the MnS phase. Figure 5b is a higher magnification image from the green box in Figure 5a. We obtain atomically resolved images of the matrix (blue rectangle), the precipitate phase (yellow rectangle), and the interface between them (red rectangle). These images reveal that the MnTe matrix (Figure 5c) and precipitated phase (Figure 5d) have different atomic arrangements. A typical interfacial boundary between the precipitate (left) and the matrix (right) is presented in a high‐resolution HAADF‐STEM image, as illustrated in Figure 5e. We can observe the atomic level of structural configuration at the interface between the nanoprecipitate and the matrix, and contrast difference between the two sides of the interface. In addition to nanorods and nanoprecipitates, high‐density dislocations and lattice distortions are found in the matrix, as shown in Figure 5f, Figures S12 and S13 (Supporting Information). These dislocations (marked with T) and lattice distortions (encircled) can be resolved in Figure 5g, which is in the Bragg‐filtered image of Figure 5f. Based on multiple high‐resolution HAADF‐STEM images, the dislocation density of the sample was determined roughly to be 3.36×10^11^ cm^−2^, as shown in Figure S12 (Supporting Information). The lattice distortion area in the sample was estimated to be 17.9% of the total (Figure S13, Supporting Information). The high‐resolution STEM images are analyzed by geometric phase analysis (GPA), which can be used to reveal a spatially distributed strain field around the dislocations and lattice distortions. The calculated phase strain distribution mapping (Figure 5h) demonstrates that large average lattice strain fluctuation can be induced, which suggests a broader distribution of static lattice strains surrounding the dislocations and lattice distortions. The creation of dislocations and lattice distortions causes a remarkable shortening in phonon relaxation time, contributing to reduced lattice thermal conductivity. Furthermore, MnS nanorods significantly suppress the lattice thermal conductivity by creating strong phonon scattering centres. Consequently, MnS nanorods combined with dislocations and lattice distortions contribute to an ultralow lattice thermal conductivity *κ*_lat_ in Mn_0.91_Ge_0.08_Sb_0.07_Te_0.9_S_0.1._

**Figure 7:**
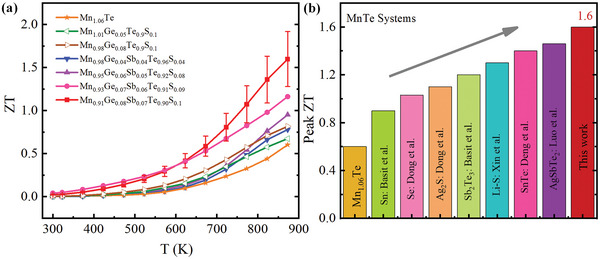
a) Temperature dependent *ZT* values of Mn_1.06‐x_Ge_x_Te_0.9_S_0.1_ and Mn_1.06‐x‐y_Ge_x_Sb_y_Te_1‐z_S_z_samples. b) Peak *ZT* comparison with other reported doped MnTe systems.

The temperature‐dependent *ZT* values for Mn_1.06‐x_Ge_x_Te_0.9_S_0.1_ and Mn_1.06‐x‐y_Ge_x_Sb_y_Te_1‐z_S_z_ samples are presented in **Figure** **7**a. *ZT* remarkably increases with the Sb—Ge—S content in the whole temperature range. Thanks to the enhanced *PF* and ultralow*κ*_lat_ induced by MnS nanorods combined with dislocations and lattice distortions, an exceptional *ZT* of ≈1.6 at 873 K was achieved in the composition of Mn_0.91_Ge_0.08_Sb_0.07_Te_0.9_S_0.1_, in contrast to ≈0.60 for pristine Mn_1.06_Te. The reported *ZT* are higher than the utmost reported MnTe systems (Figure 7b) and are competitive with other well‐known *p*‐type thermoelectric materials.^[^ ^22^, ^31^, ^32^, ^33^, ^34^, ^53^ ^]^ Such high thermoelectric performance is reproducible (Figure S14, Supporting Information). The present work indicates that environmentally friendly MnTe‐based material is a promising candidate for medium‐temperature thermoelectric materials.

## 4 Conclusion

This study proposes a new route to achieve ultralow lattice thermal conductivity and extraordinary thermoelectric performance in Mn_1.06‐x‐y_Ge_x_Sb_y_Te_1‐z_S_z_ compounds. It is found that Sb—Ge incorporation facilitates multiple valence band convergence in MnTe. Sb—Ge—S doping boosts carrier concentration and electrical conductivity. The optimized carrier concentration coupled with band structure modification results in sharp increase of *PF* 12.89 µWcm^−1^K^−2^ at 873 K in Mn_1.06‐x‐y_Ge_x_Sb_y_Te_1‐z_S_z_ series. Microstructural characterization reveals that numerous MnS nanorods are induced by S doping. Localized lattice imperfections, including MnS nanorods, dislocations and lattice distortions, were induced by Sb—Ge—S alloying, resulting in significantly reduced *κ*_lat_. Consequently, we achieve a record‐high *ZT* of 1.6 in Mn_0.91_Ge_0.08_Sb_0.07_Te_0.9_S_0.1_ at 873 K through synergistic microstructure engineering and facilitating multiple valence band convergence, making it the best MnTe‐based thermoelectric materials.

## Conflict of Interest

The authors declare no conflict of interest.

## Supporting information

Supporting Information

## Data Availability

The data that support the findings of this study are available from the corresponding author upon reasonable request.
