# Supplementary material for: Multiple Valence Bands Convergence and Localized Lattice Engineering Lead to Superhigh Thermoelectric Figure of Merit in MnTe
Source: Adv Sci (Weinh). 2023 Apr 24;10(17):2206342. doi: 10.1002/advs.202206342 (PMC10265067; doi:10.1002/advs.202206342)
Supplement: Supplementary file 1 — Supporting Information [file ADVS-10-2206342-s001.pdf]

ADVANCED SCIENCE
Open Access

Supporting Information

for *Adv. Sci.*, DOI 10.1002/advs.202206342

Multiple Valence Bands Convergence and Localized Lattice Engineering Lead to Superhigh Thermoelectric Figure of Merit in MnTe

Shahzada Zulkifal, Zhichao Wang, Xuemei Zhang, Suniya Siddique, Yuan Yu, Chong Wang, Yaru Gong, Shuang Li, Di Li, Yongsheng Zhang, Peng Wang* and Guodong Tang**

Multiple Valence Bands Convergence and Localized Lattice Engineering Lead to Superhigh Thermoelectric Figure of Merit in MnTe

Shahzada Zulkifal [1,#], Zhichao Wang[2,#], Xuemei Zhang[3] , Suniya Siddique[1], Yuan Yu[4], Chong Wang[1], Yaru Gong[1] , Shuang Li[1], Di Li[2], Yongsheng Zhang[5,*], Peng Wang,[3,*] Guodong Tang[1,*]

[1]MIIT Key Laboratory of Advanced Metallic and Intermetallic Materials Technology, School of Materials Science and Engineering, Nanjing University of Science and Technology, Nanjing 210094, PR China.

[2]Key Laboratory of Materials Physics, Institute of Solid State Physics, Chinese Academy of Sciences, Hefei 230031, China.

[3]National Laboratory of Solid State Microstructures, College of Engineering and Applied Sciences and Collaborative, Innovation Center of Advanced Microstructures, Nanjing University, Nanjing 210093, China.
[4]Institute of Physics IA, RWTH Aachen University, 52056 Aachen, Germany.
5Advanced Research Institute of Multidisciplinary Sciences, Qufu Normal University, Qufu, Shandong Province, 273165, China.

*To whom correspond should be addressed.
E-mail: tangguodong@njust.edu.cn (G. Tang)
wangpeng@nju.edu.cn
yshzhang@qfnu.edu.cn
# These authors contribute equally to this work

Table S1. Densities of undoped and doped $Mn_{1.06}Te$ samples

Sample	Density (ρ, g/cm^3)	Theoretical density (g/cm^3)
$Mn_{1.06}Te$	5.908	6.122
$Mn_{1.01}Ge_{0.05}Te_{0.9}S_{0.1}$	5.921	6.253
$Mn_{0.98}Ge_{0.08}Te_{0.9}S_{0.1}$	5.930	6.245
$Mn_{0.98}Ge_{0.04}Sb_{0.04}Te_{0.96}S_{0.04}$	5.901	6.186
$Mn_{0.95}Ge_{0.06}Sb_{0.05}Te_{0.92}S_{0.08}$	5.906	6.219
$Mn_{0.93}Ge_{0.07}Sb_{0.06}Te_{0.91}S_{0.09}$	5.922	6.251
$Mn_{0.91}Ge_{0.08}Sb_{0.07}Te_{0.90}S_{0.1}$	5.890	6.316

Table S2: Hall carrier concentration (n_H) and mobility (μ) with electrical conductivity of the samples

Sample	n_H (cm^{-3})	σ ($S\ cm^{-1}$)	μ ($cm^2\ V^{-1}\ s^{-1}$)
$Mn_{1.06}Te$	3.10E18	0.59	1.231
$Mn_{1.01}Ge_{0.05}Te_{0.9}S_{0.1}$	0.95E19	1.45	0.953
$Mn_{0.98}Ge_{0.08}Te_{0.9}S_{0.1}$	5.22E19	5.37	0.645
$Mn_{0.98}Ge_{0.04}Sb_{0.04}Te_{0.96}S_{0.04}$	8.22E19	7.71	0.586
$Mn_{0.95}Ge_{0.06}Sb_{0.05}Te_{0.92}S_{0.08}$	1.41E20	8.87	0.404
$Mn_{0.93}Ge_{0.07}Sb_{0.06}Te_{0.91}S_{0.09}$	3.52E20	19.61	0.346
$Mn_{0.91}Ge_{0.08}Sb_{0.07}Te_{0.90}S_{0.1}$	4.24E20	22.38	0.329

Table S3 Band gap and energy differences between the four valence maxima (the M, H and A points in the BZ).

eV	MnTe (PBE)	$Mn_{0.922}Ge_{0.078}Te$	$Mn_{0.922}Sb_{0.078}Te$
Band gap	0.71	1.06	0.92
$\Delta\varepsilon_{\Gamma\text{-}M}$	0.04	0.03	0.03
$\Delta\varepsilon_{\Gamma\text{-}H}$	0.26	0.24	0.27
$\Delta\varepsilon_{\Gamma\text{-}A}$	0.34	0.34	0.28

Table S4 Atomic energy levels of s, p and d states of different atoms (M=Mn, Te, Ge, Sb) calculated using all-electron full-potential method[1] (units, eV)

Atom	Valence state	s-states	p-states	d-states
Mn	$3d^5 4s^2$	-5.51		-8.10
Te	$5s^2 5p^4$	-15.45	-6.12	
Ge	$4s^2 4p^2$	-12.21	-4.34	
Sb	$5s^2 5p^3$	-13.42	-5.28	

Table S5 Defect formation energies of $MnTe_{0.891}S_{0.109}$, $Mn_{0.922}Ge_{0.078}Te$, $Mn_{0.922}Sb_{0.078}Te$ and $Mn_{0.844}Ge_{0.078}Sb_{0.078}Te$.

Doping case	ΔH_D (eV/defect)
$MnTe_{0.891}S_{0.109}$	-0.03
$Mn_{0.922}Ge_{0.078}Te$	0.35
$Mn_{0.922}Sb_{0.078}Te$	0.06
$Mn_{0.844}Ge_{0.078}Sb_{0.078}Te$	0.39

Calculation details of the formation energy of S, Ge, and Sb doping

The defect formation energy of S, Ge and Sb doping in MnTe using the following formula,

$$\Delta H_D = [E_D - E_H] + \sum_i n_i \mu_i,$$

where E_D and E_H are the total energies of supercells with and without defects, respectively. n_i is the number of element i: $n_i < 0$ and $n_i > 0$ represent atoms are added to or removed from the host supercell. μ_i is the corresponding chemical potentials of each element [2]. For Ge/Sb/S in MnTe, the chemical potential of each element is determined based on the formed possible equilibrium region using all related Ge-Sb-Mn-Te or S-Mn-Te compounds, which are taken from the Open Quantum Materials Database[3]. When Ge-Sb co-doping in the MnTe, the equilibrium region is surrounded by the Mn, $MnTe_2$, Mn_3Ge and Sb compounds, and

$$\mu_{Mn} = E(Mn)$$

$$\mu_{Mn} + 2\mu_{Te} = E(MnTe_2)$$

$$3\mu_{Mn} + \mu_{Ge} = E(Mn_3Ge)$$

$$\mu_{Sb} = E(Sb)$$

The Mn, $MnTe_2$ and MnTeS compounds enclose the equilibrium region of S doping in MnTe, and

$$\mu_{Mn} = E(Mn)$$

$$\mu_{Mn} + 2\mu_{Te} = E(MnTe_2)$$

$$\mu_{Mn} + \mu_{Te} + \mu_S = E(MnTeS)$$

With these determined chemical potentials, we can calculate the defect formation energies of $MnTe_{0.891}S_{0.109}$, $Mn_{0.922}Ge_{0.078}Te$, $Mn_{0.922}Sb_{0.078}Te$ and $Mn_{0.844}Ge_{0.078}Sb_{0.078}Te$ in Table S5.

Fig. S1. Crystal structures of (a) pristine MnTe, (b) $MnTe_{0.891}S_{0.109}$ and $Mn_{0.844}Ge_{0.078}Sb_{0.078}Te$. The purple, royal blue, green and orange spheres represent Mn, Te and Ge and Sb atoms, respectively.

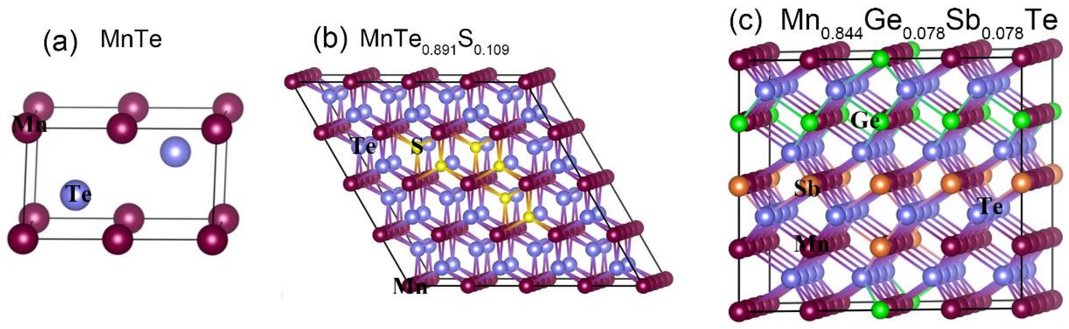

We construct a (4 × 4 × 2) MnTe supercell (*a* = 16.58 Å, *b* = 16.58 Å, *c* = 13.42 Å, containing 64 cations and 64 anions) based on its crystal structure. Taking $MnTe_{0.89}1S_{0.109}$ as an example, the experimentally suggested S doping concentration is ~10%, which means that we should substitute 7 Te atoms by S in the supercell. Instead of randomly substituting all these Te by S, we gradually replace Te by S one by one and find the lowest energy defect configuration each time: replacing one Te with one S atom and optimizing it; based on the one-S configuration, using another S atom to substitute all possible Te positions and finding the lowest energy two-S configuration; keeping this going on until 7 S substituted into Te positons. The similar procedure is used in setting up the $Mn_{0.844}Ge_{0.078}Sb_{0.078}Te$ supercell.

Fig. S2. SEM image and Energy Dispersive Spectrometer (EDS) elemental mapping image for $Mn_{0.91}Ge_{0.08}Sb_{0.07}Te_{0.90}S_{0.1}$.

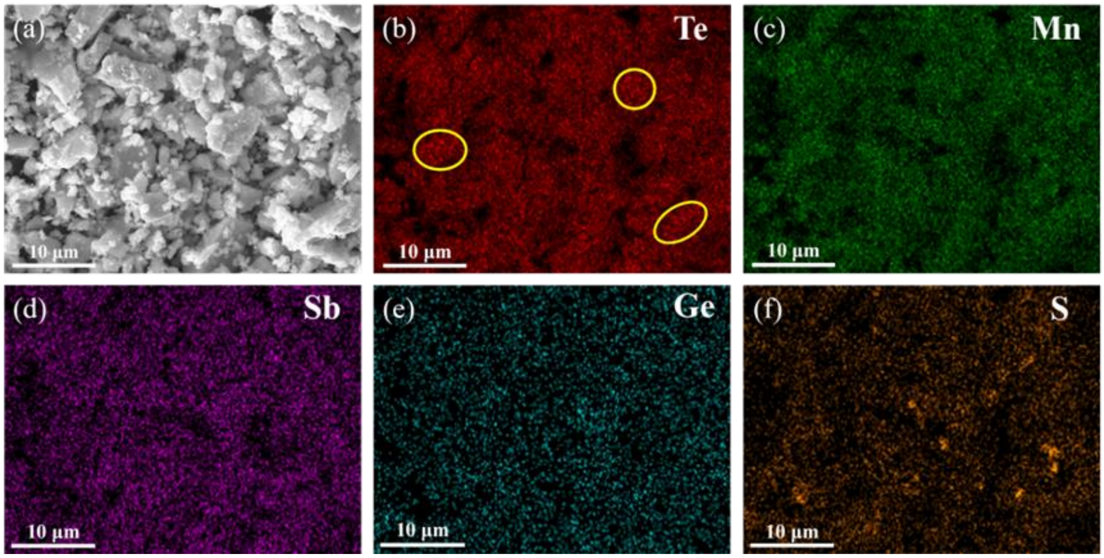

Fig. S3. Enlarged XRD plot of narrow angle from 43°-44.5°, of undoped and Sb-Ge-S-doped $Mn_{1.06}Te$ samples.

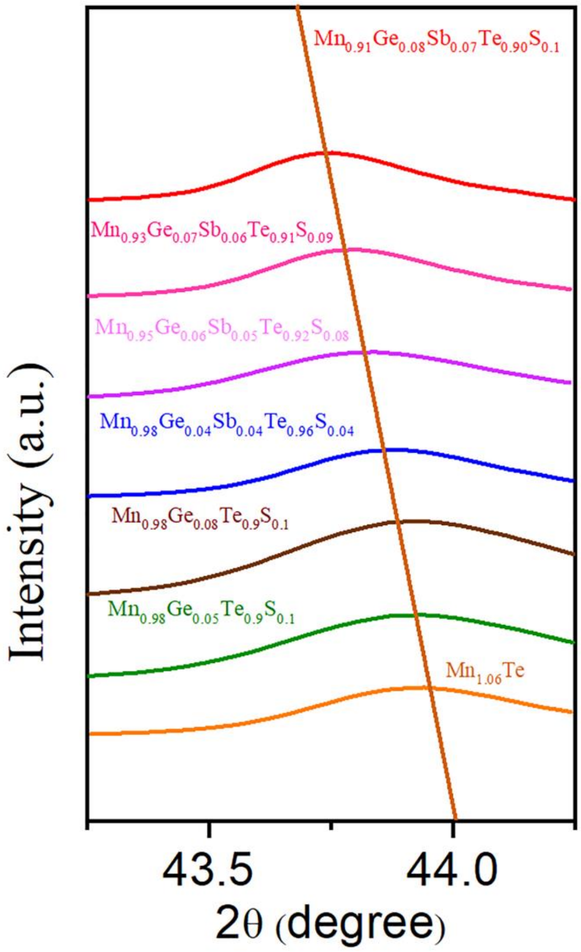

Fig. S4. (a) SEM of polished surface for $Mn_{0.91}Ge_{0.08}Sb_{0.07}Te_{0.90}S_{0.1}$, (b-d) Elemental mapping of $Mn_{0.91}Ge_{0.08}Sb_{0.07}Te_{0.90}S_{0.1}$ taken from the area in (a).

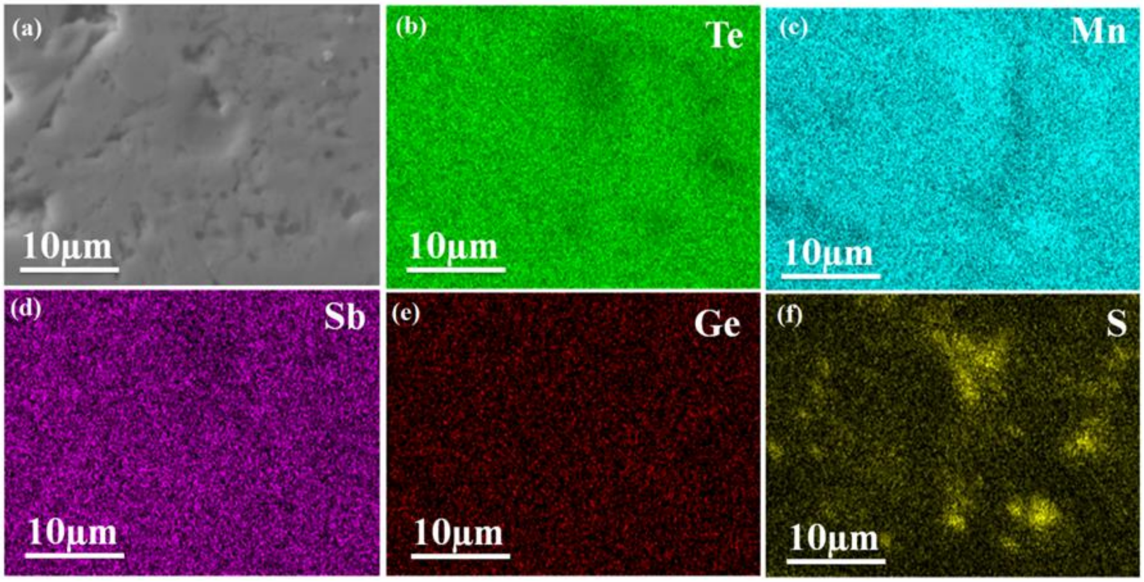

Fig. S5. The modified PBE-calculated electronic band structures of pristine MnTe using the HSE calculations, suggested in ref.[4]

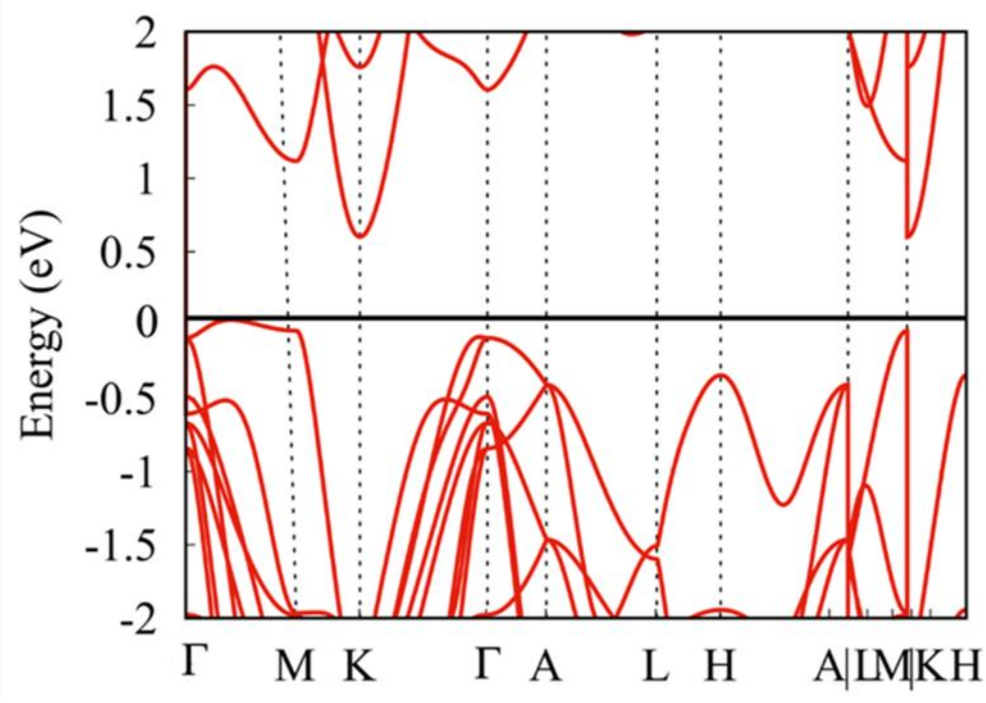

Fig. S6. (a) Projected density of states (PDOS) and (b) crystal orbital Hamilton population (COHP) analysis for pristine MnTe.

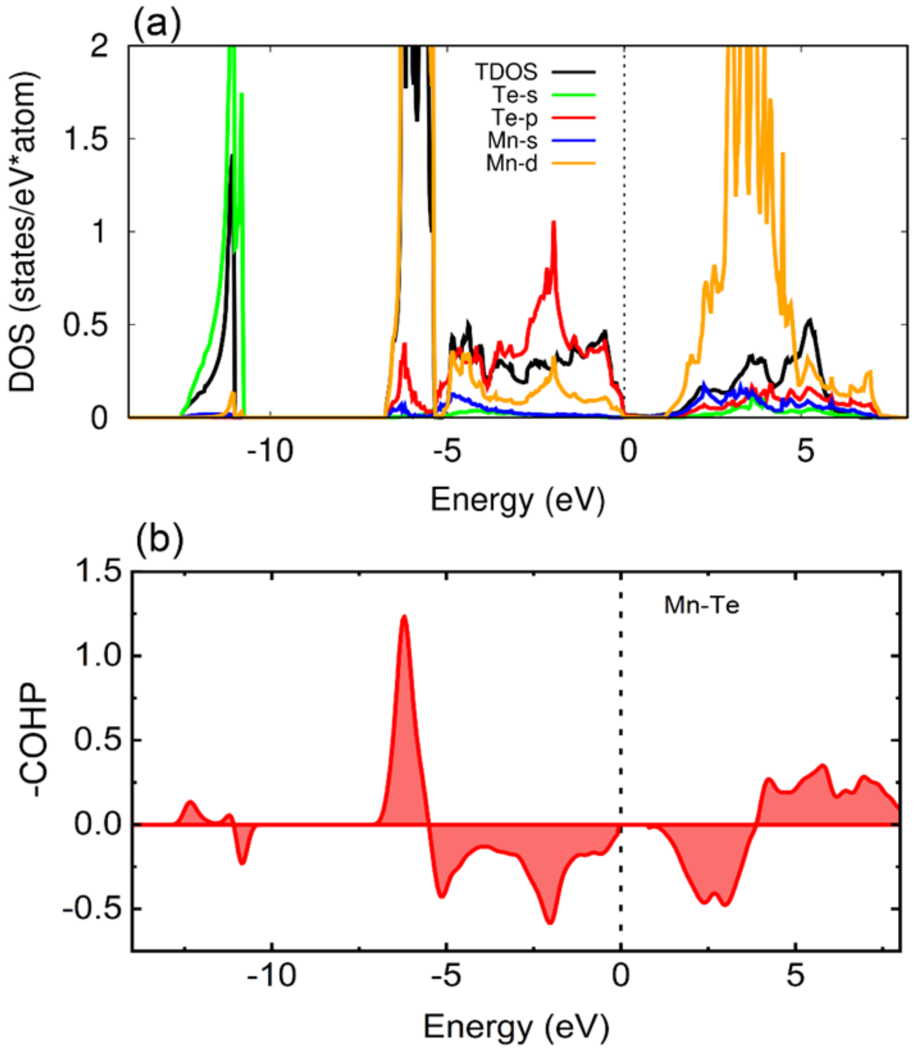

Fig. S7. Crystal structures of (a) $Mn_{0.922}Ge_{0.078}Te$ and (b) $Mn_{0.922}Sb_{0.078}Te$. The purple, royal blue, green and orange spheres represent Mn, Te and Ge and Sb atoms, respectively. Electronic band structures of (c) $Mn_{0.922}Ge_{0.078}Te$ and (d) $Mn_{0.922}Sb_{0.078}Te$. The scale bar is the magnitude of the spectral weight, which characterizes the probability of the primitive cell eigenstates contributing to a particular supercell eigenstates of the same energy.

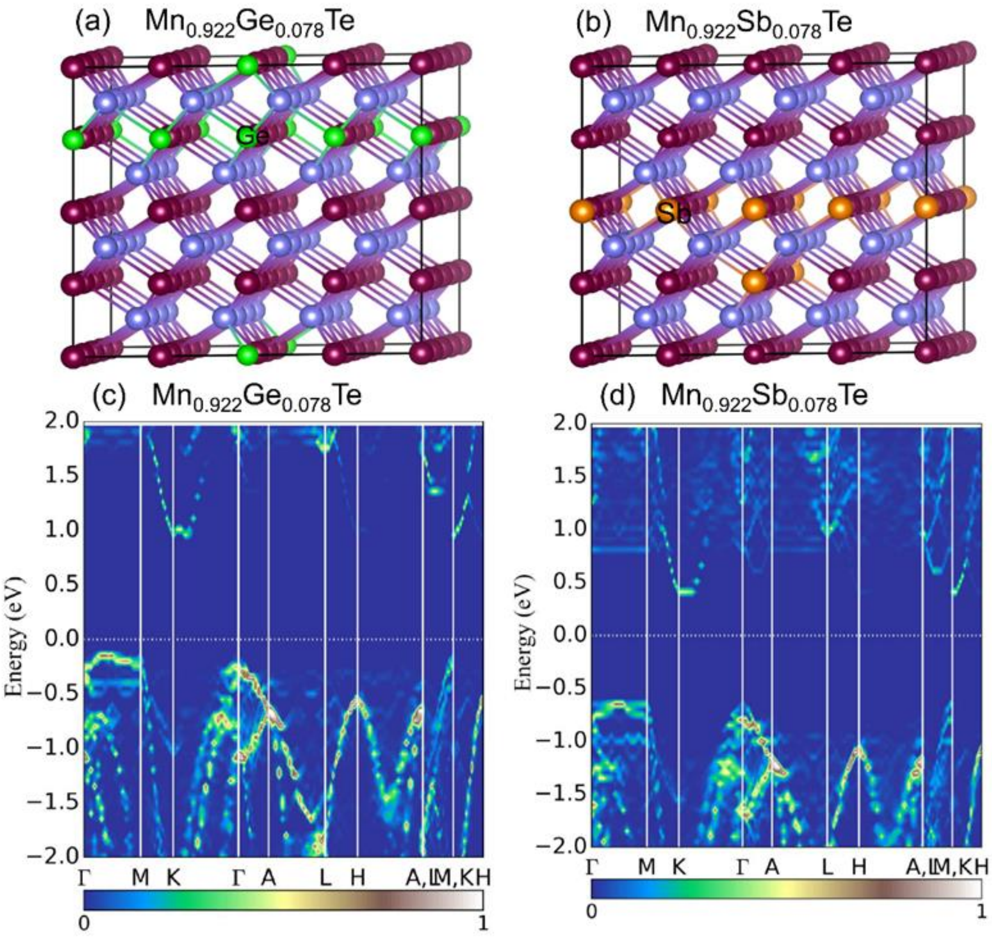

Fig. S8. Electrical conductivity (κ_{ele}) of $Mn_{1.06-x}Ge_xTe_{0.9}S_{0.1}$ and $Mn_{1.06-x-y}Ge_xSb_yTe_{1-z}S_z$ samples.

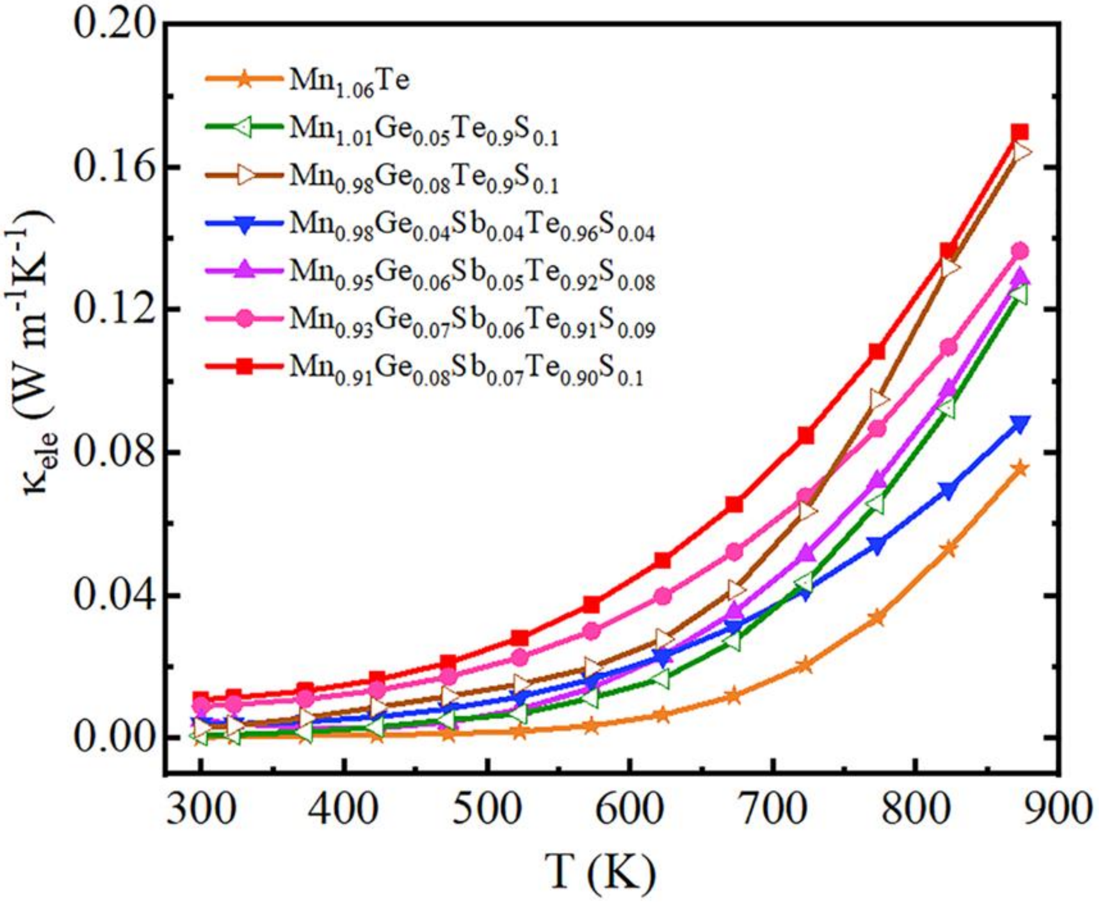

Fig. S9. Lorenz number (L) as a function of temperature for $Mn_{1.06-x}Ge_xTe_{0.9}S_{0.1}$ and $Mn_{1.06-x-y}Ge_xSb_yTe_{1-z}S_z$ samples

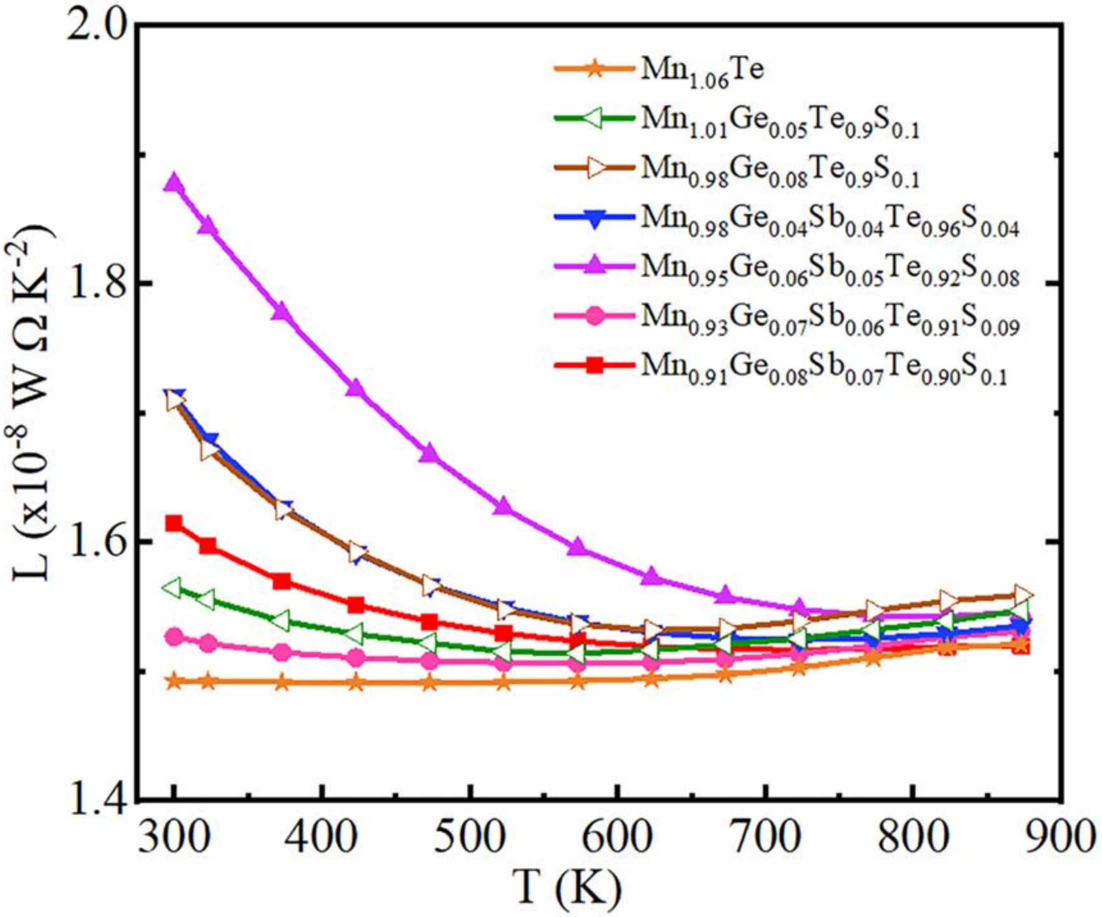

Fig. S10. Thermal diffusivity (*D*) of $Mn_{1.06-x}Ge_xTe_{0.9}S_{0.1}$ and $Mn_{1.06-x-y}Ge_xSb_yTe_{1-z}S_z$ samples

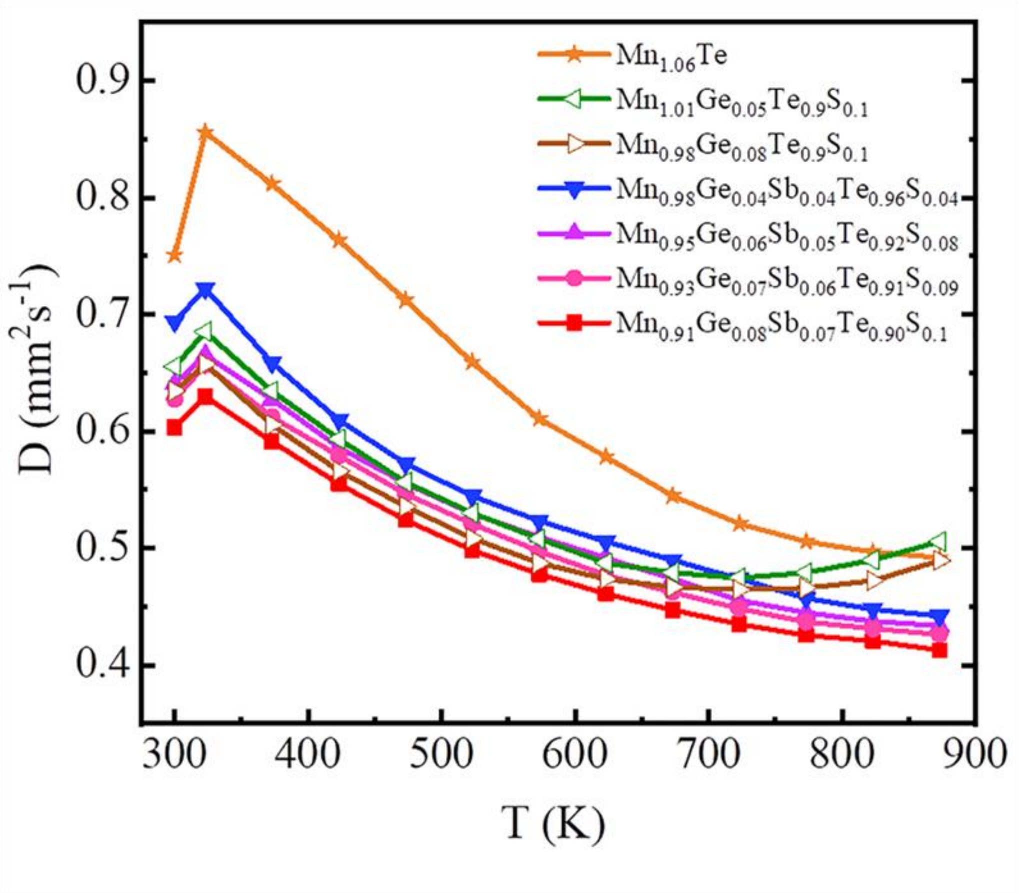

Fig. S11. STEM-EDS elemental mapping of quasi-circular nano-precipitates reveals composition of these precipitated phases is mainly Mn and S elements

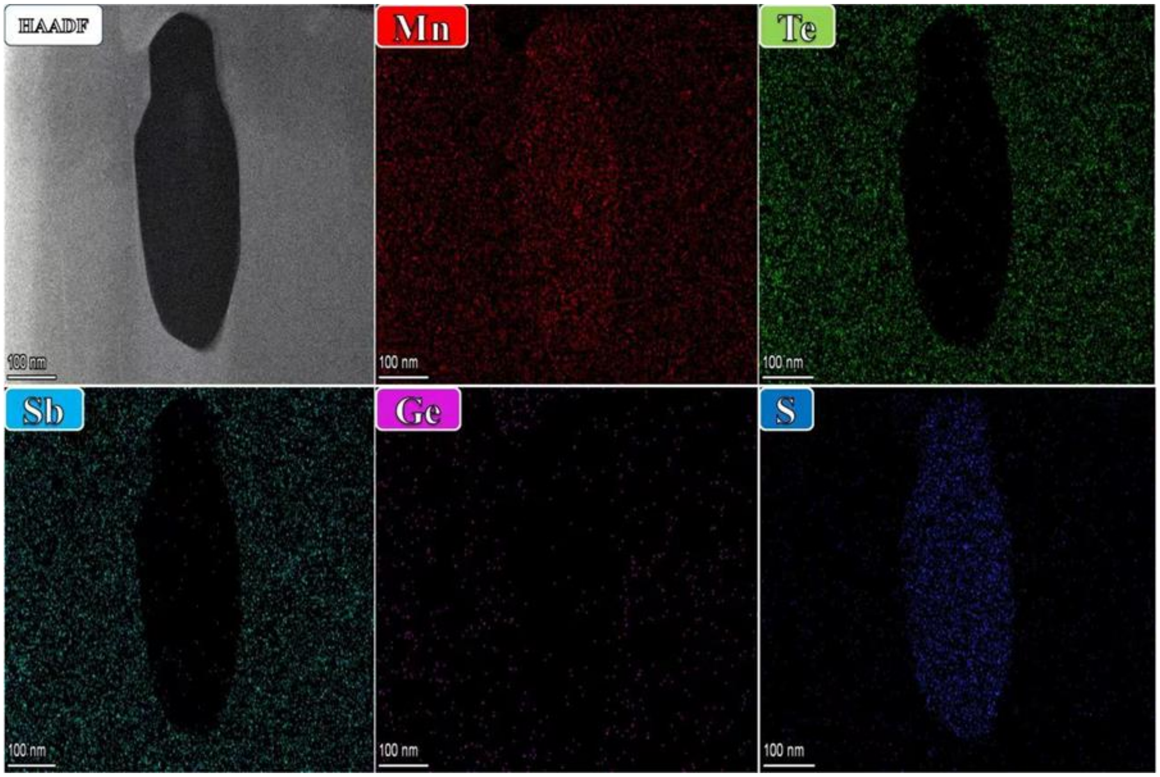

Fig. S12. (a, c)HAADF-STEM images of $Mn_{0.91}Ge_{0.08}Sb_{0.07}Te_{0.90}S_{0.1}$; (c,d) the corresponding filtered image based on (a) and (c) showing dislocations (marked with T).

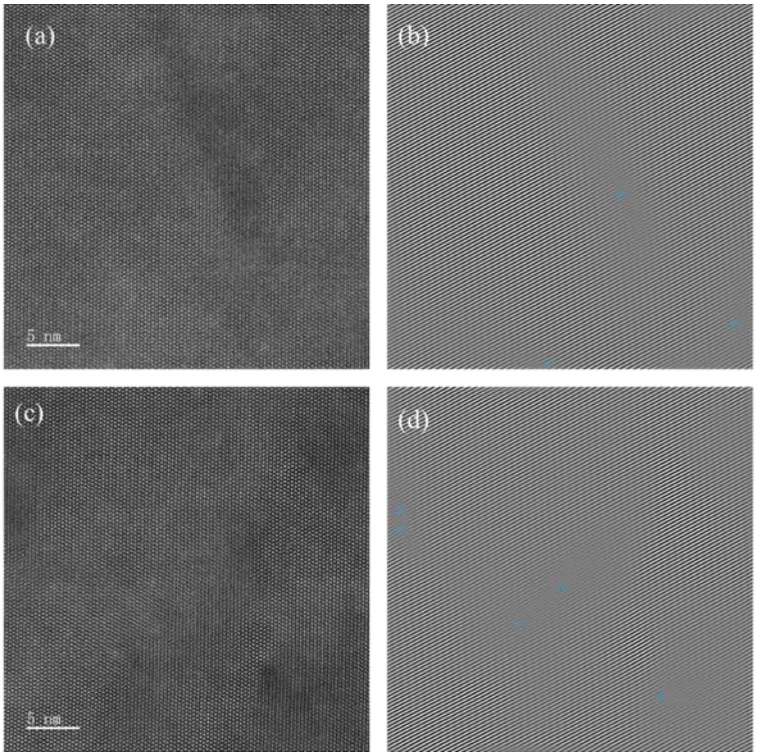

Fig. S13. $Mn_{0.91}Ge_{0.08}Sb_{0.07}Te_{0.9}S_{0.1}$ with distortions: (a) HAADF-STEM image; (b) the enlarged view of area in red box of (a); (c) FFT image of (b), which shows spliting of the diffraction spots, indicating large lattcie distortions in the matrix; (d) HAADF-STEM image of another area with lattice distortions.

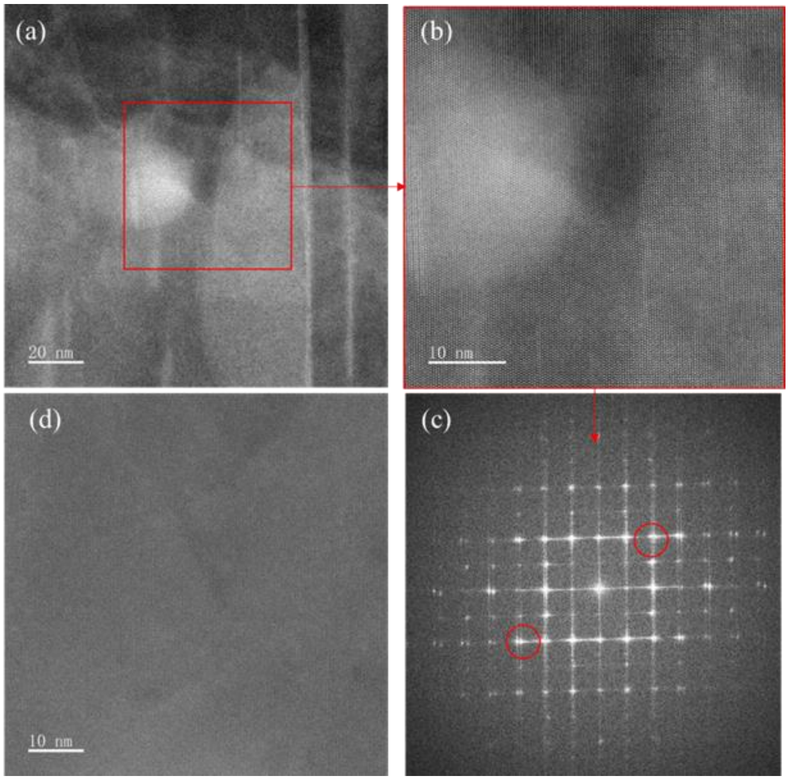

Fig. S14. Repeat measurement results of (a) electrical conductivity (σ), (b) Seebeck coefficient (S), (c) thermal conductivity (κ_T), (d) ZT for $Mn_{0.91}Ge_{0.08}Sb_{0.07}Te_{0.9}S_{0.1}$

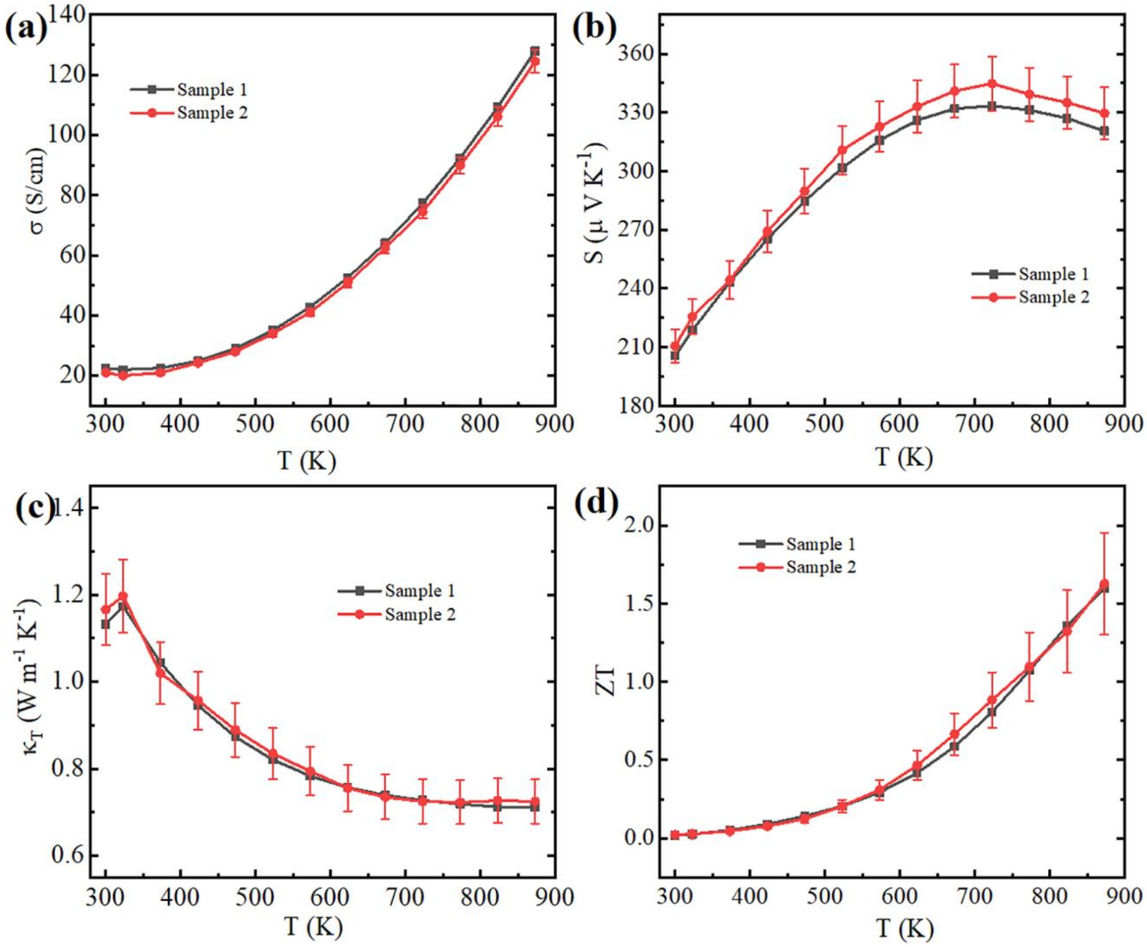

Fig. R2 The temperature dependent Hall carrier concentration and mobility for $Mn_{0.95}Ge_{0.06}Sb_{0.05}Te_{0.92}S_{0.08}$.

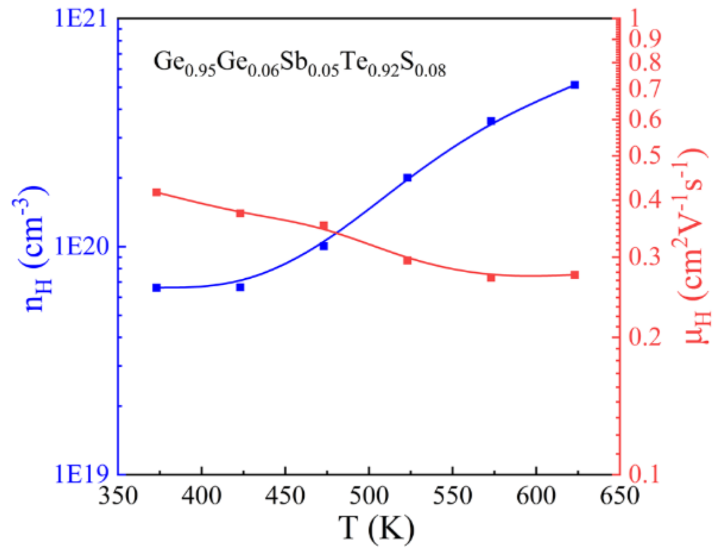

References

1. K. Schwarz, P. Blaha, *Comput. Mater. Sci.* **2003**, *28*, 259.

2. X. M. Zhang, M. Y. Toriyama, J. P. Male, Z. Z. Feng, S. P. Guo, T. T. Jia, Z. Y Ti, G. J. Snyder, Y. S. Zhang, Mater. Today Phys., **2021**, *19*, 100415.

3. S. Kirklin, E S. James, M. Bryce, T. Alex, W D. Jeff, A. Muratahan, R. Stephan, W. Chris, *npj Comput. Mater.* **2015**, *1*, 15010

4. H. Deng, X. Lou, W. Lu, J. Zhang, D. Li, S. Li, Q. Zhang, X. Zhang, X. Chen, D. Zhang, Y. Zhang, G. Tang, *Nano Energy* **2021**, *81*, 105649.
